# *Trypanosoma cruzi* Infection and Host Lipid Metabolism

**DOI:** 10.1155/2014/902038

**Published:** 2014-09-03

**Authors:** Qianqian Miao, Momar Ndao

**Affiliations:** ^1^National Reference Centre for Parasitology, Research Institute of McGill University Health Centre, Montreal General Hospital, Montreal, QC, Canada H3G 1A4; ^2^Department of Microbiology and Immunology, McGill University, Montreal, QC, Canada H3A 2B4

## Abstract

*Trypanosoma cruzi* is the causative agent of Chagas disease. Approximately 8 million people are thought to be affected worldwide. Several players in host lipid metabolism have been implicated in *T. cruzi*-host interactions in recent research, including macrophages, adipocytes, low density lipoprotein (LDL), low density lipoprotein receptor (LDLR), and high density lipoprotein (HDL). All of these factors are required to maintain host lipid homeostasis and are intricately connected via several metabolic pathways. We reviewed the interaction of *T. cruzi* with each of the relevant host components, in order to further understand the roles of host lipid metabolism in *T. cruzi* infection. This review sheds light on the potential impact of *T. cruzi* infection on the status of host lipid homeostasis.

## 1. Introduction 

*Trypanosoma cruzi* (*T. cruzi*) is the etiological agent of Chagas disease (CD). It is estimated that 8 million people are infected worldwide [1]. In the endemic area of South and Central America, CD is transmitted through contact with the feces of the triatomine bug (the kissing bug). When taking a blood meal from a human, the bug defecates on the skin where* T. cruzi* can enter the wound or the mucosal membrane by scratching. Effective vector-control programs have greatly decreased disease transmission in these areas [2, 3]. However, CD was brought to North America, Europe, and Asia by infected individuals, through migration in recent years. In nonendemic area, CD is transmitted through blood transfusion, organ transplantation, and congenital transmission [4].

During the* T. cruzi *infection process, the parasite interacts with a wide range of host immunological and metabolic factors. In the past decade, special attention was given to the close relationship between* T. cruzi *infection and host lipid metabolism. Several research groups have uncovered the interaction between* T. cruzi *and players in the host cholesterol transport and storage system such as macrophage [5–7], adipocytes [8], low density lipoprotein (LDL), and high density lipoprotein (HDL) [9–11]. The molecular landscape and impact of these relationships in* T. cruzi *infection and pathogenesis, as well as host immunological responses and inflammatory reactions, will be reviewed in this paper.

There are three stages in CD progression: acute, indeterminate, and chronic. Although the majority of infected individuals are asymptomatic while carrying the life-long infection, some develop severe symptoms upon infection. During the acute stage, infected individuals may develop unspecific symptoms such as fever, nausea, diarrhea, and rash, as well as severe symptoms such as a raised inflammatory lesion at the site of parasite entry (chagoma), unilateral periorbital edema (Romana's sign), lymphadenopathy, and hepatosplenomegaly [12]. The majority of patients survive the acute stage and enter the prolonged indeterminate stage without overt symptoms of disease, which lasts for life. However, thirty percent of patients develop chronic CD, which includes grave symptoms such as megaesophagus, megacolon, and chronic heart disease [13].

*T. cruzi *has a complex life cycle and undergoes several transformations during the infection process. The parasite exists mainly in its epimastigote form in the triatomine vector. It transforms into metacyclic trypomastigote in the hind gut of the vector which is then defecated to infect human host. Once in the host, the metacyclic form infects a wide range of phagocytic (i.e., monocytes, neutrophils, mast cells, and macrophages) and nonphagocytic cells (i.e., epithelial cells, endothelial cells, fibroblasts, and mesenchymal cells). Upon infection, trypomastigotes transform into intracellular amastigotes and divide by binary fission. Once the division process is complete, amastigotes transform back into blood trypomastigotes which escape the cell to infect neighbouring cells or enter the blood circulation [14].

## 2. *T. cruzi* Infection and Macrophage Lipid Bodies 

Lipid bodies (LB), also named lipid droplets or adiposomes, are lipid-rich organelles existing in almost all organisms. Unlike other organelles, lipid bodies are uniquely surrounded by a monolayer of phospholipids [15]. The core of the lipid body is rich in neutral lipids, mainly triacylglycerol and sterol esters, as well as other putative membranous structures [15]. Historically, lipid bodies were thought to function in neutral lipid storage and transport; however, recent research has uncovered their importance in regulation of host immune responses. Lipid bodies are involved in the formation of paracrine mediator eicosanoids in cells involved in inflammatory processes [16, 17]. The number of lipid bodies in leukocytes increases in response to a variety of inflammatory conditions, such as atherosclerosis and mycobacterial infections [18, 19].

**Figure 1:**
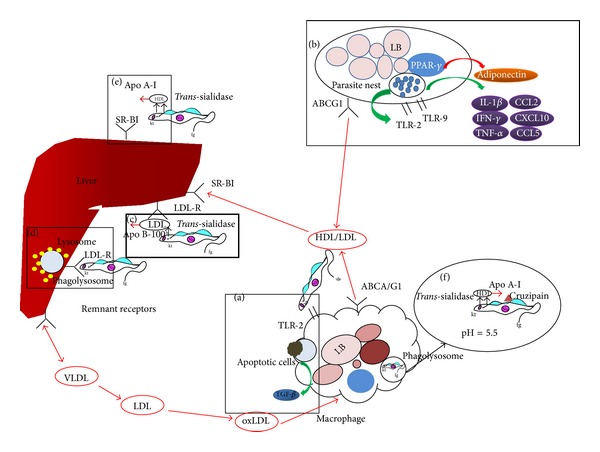
*Trypanosoma cruzi *interacting with various components of host lipid metabolism. (a)* T. cruzi *infects macrophage and activates TLR-2 signaling which causes the increased lipid bodies (LB) number and altered LB morphology. This effect is further enhanced by macrophage uptake of apoptotic cells. Increased LB number causes increased eicosanoid production, which leads to upregulation of TGF-*β* and increased cell susceptibility to* T. cruzi *infection. (b)* T. cruzi *uses adipocytes as a reservoir for chronic infection.* T. cruzi *infection causes adipocytes to display an inflammatory phenotype, upregulating cytokines such as IL-1*β*, IFN-*γ*, TNF-*α*, CCL2, CXCL10, and CCL5. TLR-2 and 9, which are essential to* T. cruzi *infection, are also upregulated. The infection also causes downregulation of adiponectin secretion via PPAR-*γ* expression. (c) Host LDL inhibits* T. cruzi trans*-sialidase and increases* T. cruzi *infection* in vitro*. LDL can be taken up by the liver and extrahepatic cells by LDLR. It is unknown whether LDL-LDLR interaction plays a role in* T. cruzi *infection. (d)* T. cruzi *enters host cell via LDLR. LDL-R activation leads to lysosomal recruitment to parasitophorous vacuole and parasite internalization. (e) HDL inhibits* T. cruzi trans*-sialidase activity and increases* T. cruzi *infection* in vitro*. HDL is uptaken by host cells via receptor mediated interaction with SR-BI. Whether this interaction can be utilized by* T. cruzi *cell entry process is not known. (f) Apo A-I in the HDL complex is cleaved by the major cysteine protease of* T. cruzi, *cruzipain. Cruzipain and* trans*-sialidase are similarly expressed and located during different life stages of* T. cruzi*. It is possible that HDL is bound to the surface of* T. cruzi *trypomastigotes by* trans*-sialidase and is cleaved by cruzipain in the acidic environment in the parasitophorous vacuole. Host cholesterol transport by VLDL, LDL, and HDL is indicated in red arrows. Host VLDL and lipid-poor nascent HDL particles are produced in the liver. By effluxing cholesterol, host VLDL transforms to become LDL and nascent HDL becomes mature HDL. LDL particles can be oxidized and uptaken by macrophage. Lipid-laden macrophages are termed foam cells and are major contributors in host atherosclerosis development. HDL effluxes cholesterol from peripheral tissue via the action of ABCA1 or ABCG1 and returns cholesterol to hepatic tissues for storage or excretion.

During acute* T. cruzi *infection, host macrophages are strongly activated and will inhibit parasite replication [20]. It has been demonstrated that activated murine macrophages are capable of killing the parasites* in vitro* [5–7]. The macrophage inhibition of parasite replication also correlated positively with increases in the oxidative burst activity [21], tumor necrosis factor-alpha production (TNF-*α*) [7], and nitric oxide secretion [22]. Macrophages from more resistant C57/BL6 mice strain also secreted higher TNF-*α* in the* in vivo* experiments compared to macrophages from the susceptible strains, such as C3H and BALB [23]. In macrophage-depleted* T. cruzi *infected rats, myocardial parasite load as well as blood parasitemia was significantly increased compared to control [24]. When irradiate rats, which have very low numbers of T and B lymphocytes, were treated with recombinant Interferon-*γ* (IFN-*γ*), which classically activates host macrophages,* T. cruzi *parasite load was significantly reduced [25]. These findings demonstrated the importance of macrophage in the clearance of parasites. However, the roles of macrophage in* T. cruzi *infection may not be as simple as previously thought. Certain features of macrophage activation may aid in parasite survival in the host. Melo showed that, during acute* T. cruzi *infection, there is a prominent increase in the number of lipid bodies in macrophages [26]. This increase in lipid body formation correlated with increased parasite load* in vivo* [27]. It was further demonstrated that the induction of lipid body formation during* T. cruzi *infection was Toll-like receptor (TLR-2) dependent and was enhanced by the uptake of apoptotic cells, which causes macrophage to interact with *α*_*v*_ *β*3 integrin and activates TGF-*β*-dependent lipid body formation [27, 28]. Increased levels of TGF-*β* are known to cause phagocytic cells to become permissive to* T. cruzi *infection [29, 30] (Figure 1(a)).

Increased lipid body formation also led to increased eicosanoid prostaglandin E_2_  (PGE_2_) production in inflammatory macrophages. Prostaglandins are known to inhibit TNF-*α* and IFN-*γ* production, while enhancing TGF-*β* secretion [31–33]. Release of prostaglandins reduces macrophage trypanocidal function [31, 34]. Although the impact of PGE_2_ release in* T. cruzi *infection is contradictory, the release of PGE_2_ was correlated in resistance against certain strains of* T. cruzi *infection [35]. In addition, treatment with nonsteroidal anti-inflammatory drugs (NSAIDs) or cyclooxygenase (COX) inhibitors was able to modulate lipid body formation and decrease PGE_2_ production, which led to decreased parasite growth in macrophages [27, 36].

Furthermore, these newly formed lipid bodies also varied significantly in size and light density, which indicated the structural participation of these organelles in immune responses to* T. cruzi *infection. The structural alterations of LB in macrophages may be related to the different lipid compositions in the organelle, stage of new LB formation, or fluctuation of the arachidonate production and concentration [37]. Ultrastructural investigation revealed that the newly formed lipid bodies are localized in close proximity to macrophage phagolysosomes or even within these structures. This suggests that lipid bodies may interact with the phagolysosomes during acute* T. cruzi *infection [38]. Lipid bodies are known to provide nutrients to intracellular parasites such as* Leishmania chagasi, *which are located in the phagolysosome [39]. The relationship between lipid body and phagolysosome can also be beneficial to the host. As reviewed by Melo et al., lipids recruited during lipid body formation, such as arachidonic acid (AA), are able to activate actin assembly, phagosome-lysosome fusion, and phagosome maturation [40, 41]. In addition, these lipids can activate phagosomal nicotinamide adenine dinucleotide phosphate- (NADPH-) oxidase, which leads to pathogen elimination [42]. The implication of the close localization of lipid bodies and phagolysosome in* T. cruzi *infected macrophages needs to be further investigated.

## 3. *T. cruzi* Infection and Host Adipose Tissue 

Adipose tissue is one of the largest organs in the host. It is comprised of a wide range of cell types including adipocytes, pericytes, monocytes, macrophages, and endothelial cells [43]. The function of adipose tissue has long been considered to be energy storage. More than 95% of adipocyte cell mass is lipid droplets where triglycerides and cholesterol esters are stored [44]; however, it was recently uncovered that the functions of adipose tissue include not only energy storage, but also metabolic regulation, neuroendocrine, and immune regulations [45]. Adipose tissue is home to a variety of adipokines, such as adiponectin [46], leptin [47], and resistin [48], which are prominent regulators of lipid homeostasis and immunological functions.

Recently, metabolic dysfunction was linked to CD pathogenesis by the observation that there are greater incidences of diabetes in* T. cruzi *infected individuals [49]. Later research showed decreased insulin level and dysregulated glucose responses among CD patients [50, 51], which further demonstrated the dysregulation of energy metabolism in these patients. It was also shown that chemically induced diabetic mice as well as genetically predisposed *db*/*db* diabetic mice with defective leptin receptors had higher parasitemia and mortality after* T. cruzi *infection, which suggests that the dysregulation of host metabolism may be beneficial to parasitic survival in the host [52].

Adipocytes are the key cell type in metabolic dysregulations such as diabetes [53]. The role of adipocytes in CD pathogenesis was therefore investigated. Mice infected with* T. cruzi *showed symptoms of hypoglycemia during the acute stage of infection; however, insulin sensitivity was unaltered [54]. Levels of adiponectin and leptin were significantly reduced in* T. cruzi *infected mice, which further suggest the altered state of glucose regulation and possible adipocyte involvement in disease progression [54]. Adiponectin is the only adipokine secreted exclusively by adipocytes and is strongly associated with insulin resistance and hyperglycemia. High parasite load was detected in the adipose tissue at the chronic stage, 300 days postinfection, as measured by quantitative polymerase chain reaction (qPCR). Decreased levels of adiponectin in the plasma and adipose tissue of infected mice were also observed during the chronic stage. Microscopic investigation revealed the preferred localization of* T. cruzi *in the brown fat of adipose tissue, where lipid bodies are higher in number and smaller in size compared to white adipocytes. These findings suggest that adipose tissues may serve as the parasitic reservoir during chronic infection and adipokine synthesis was disrupted possibly due to the infection [54]. Observations that* T. cruzi *parasite is present in the adipose tissue biopsy of chronically infected human patients have further confirmed the finding that adipose tissue is the reservoir of chronic* T. cruzi *infection [55]. Several follow-up studies have also shown the susceptible nature of adipocytes to* T. cruzi *infection [8, 56].

*In vitro* infection of cultured adipocytes with* T. cruzi *revealed that a panel of proinflammatory cytokines was upregulated; these include IL-1*β*, IFN-*γ*, TNF-*α*, chemokine ligand (CCL2), CCL5, and C-X-C motif chemokine 10 (CXCL10) The expressions of TLR-2 and 9 are also upregulated [8]. Other pathways, such as notch, extracellular signaling-regulated kinases (ERK), and phosphoinositide-3-kinases (PI3K), were also activated. It was shown that both ERK and PI3K pathways were activated upon* T. cruzi* infection [57, 58]. Furthermore, PPAR-*γ* is highly expressed in adipose tissue and, along with adiponectin, exerts anti-inflammatory effect [59]. Levels of peroxisome proliferator-activated receptor (PPAR-*γ*) were decreased in the infected cells, which may have led to the decreased secretion of adiponectin and increased inflammatory reactions. These findings suggest that infection of adipocytes with* T. cruzi *may contribute to the systemic proinflammatory immune responses as well as metabolic dysregulation [8] (Figure 1(b)).

In summary, recent research has revealed that adipose tissue may be the most important reservoir for* T. cruzi *chronic infection and these infected adipocytes display a proinflammatory phenotype. Altered activation profile of several kinase pathways in adipose tissues may also contribute to host metabolic dysregulation. However, questions remain unanswered. It is clear that chronic* T. cruzi *infection displays tissue tropism; however the evolutionary benefits of* T. cruzi *residing in adipocytes are unknown.* T. cruzi *may utilize the lipid stores within the adipocytes for its multiplication and survival. It is also possible that* T. cruzi *chooses adipocytes for its prolonged life-span. In addition, the specific mechanism of* T. cruzi*-adipocyte interaction is unknown. Further research is needed to unravel the biological processes behind the relationship between* T. cruzi *and adipocytes.

## 4. *T. cruzi* Infection and Host Cholesterol Transport Pathways

*T. cruzi *glycoprotein 85 (gp85)/*trans*-sialidase is similar to that of viral and bacterial neuraminidases. However, unlike other neuraminidases, upon hydrolysis of *α*-linked sialic acid from glycoconjugates on cell surfaces,* T. cruzi trans*-sialidase transfers the sialic acid onto parasitic receptors [60]. The expression and activity of* trans*-sialidase are developmentally regulated and are present at about the same extent in epimastigotes and trypomastigotes. Minimal* trans*-sialidase activity was detected in amastigotes [61].* Trans*-Sialidase is known to be involved in trypomastigote cell adhesion and invasion process by interacting with a wide range of ligands, such as laminin, fibronectin, and collagen [62–65]. Inhibition of* T. cruzi trans*-sialidase by specific antibodies led to the increased rate of infection [66].

Cholesterol transport chains are the major components of maintaining host lipid homeostasis and lipoproteins are essential players in these pathways. Lipoproteins are categorized based on their density and protein content into high density lipoproteins (HDL, density 1.603–1.210), low density lipoproteins (LDL, density 1.019–1.603), intermediate density lipoproteins (IDL, density 1.006–1.019), very low density lipoproteins (VLDL, density 0.95–1.006), and chylomicrons (density < 0.95). All lipoproteins allow the transport of hydrophobic lipid contents, such as cholesterol, triglycerides, and phospholipids, within the hydrophilic blood circulation system.

LDL is characterized by the presence of a single copy of apolipoprotein B-100 (Apo B-100) molecule on its surface. It is generated from liver-derived VLDL by a process mediated by lipoprotein lipase and hepatic lipase as well as lipid exchange proteins [67, 68]. LDL has been shown to be a potent inhibitor of* T. cruzi trans*-sialidase and enhances the infection of human fibroblasts* in vitro *in a dose-dependent manner [10]. The enhanced infection rate seen upon the addition of LDL* in vitro* is comparable to that of the enhancement caused by* trans*-sialidase inhibition [10]. LDL particles were seen covering the parasite cellular surface of* T. cruzi *trypomastigotes, but not amastigotes [69]. The localization of LDL particles correlates with* trans*-sialidase localization on the parasite surface and suggests that LDL may directly inhibit* T. cruzi *surface* trans*-sialidase to enhance rate of infection (Figure 1(c)). However, the exact molecular mechanism of this interaction has yet been demonstrated.

Previous reports have also shown that LDL can be endocytosed by* T. cruzi* epimastigotes [69]. Gold-labelled LDL particles were found within flagellar pockets. Immunoelectron microscopy showed that* trans*-sialidase expression is most concentrated in the flagellar pocket region, which suggested that despite LDL inhibition of* T. cruzi trans*-sialidase,* trans*-sialidase may also facilitate LDL endocytosis by the parasite [70]. Reservosomes are the site of accumulated endocytosed proteins and lipids in* T. cruzi*. This organelle in the parasite provides support for metacyclogenesis from epimastigotes to trypomastigotes [71, 72]. LDL particles were also found in the* T. cruzi *membrane enclosed vesicles and reservosome within the parasite. LDL may be stored and processed in the reservosome for usage during this transformation and infection process [73]. Similar process of LDL uptake was also demonstrated in* Leishmania amazonensis*, a parasite closely related to* T. cruzi *in the Trypanosomatidae family [74].

Another important molecule in the LDL metabolic cycle is the LDL receptor (LDLR). LDLR plays an essential role in the internalization of circulating LDL in the host liver and peripheral cells. A significant amount of cholesterol is delivered to these organs via the interaction of LDL-LDLR [75]. Approximately 50% of LDL is removed at the liver [76]. LDLR also facilitates the endocytosis of a variety of other ligands, such as proteinases and proteinase-inhibitor complexes, as well as interacting with cytoplasmic adaptor proteins which have signaling transduction functions [77]. The expression of LDLR by the host cell is regulated by a wide range of lipid metabolic and immune regulatory stimuli, such as intracellular cholesterol level, oxysterols, various growth factors, and cytokines [78, 79]. Ruan et al.  demonstrated that, in human mesangial cells, increased levels of TNF-*α*, TGF-*β*, and IL1-*β* caused increased transcription of LDLR [80]. LDLR was previously shown to be a potential host receptor for Hepatitis C virus (HCV) and other flaviviridae viruses [81, 82]. However, this direct interaction was not documented in parasitic infections until recently.

The* T. cruzi* parasite specifically binds to LDLR during the infection process [83]. Activation of LDLR facilitates the recruitment of lysosomes to the parasitophorous vacuole, which leads to the internalization of* T. cruzi *into the cytoplasm. Disruption of LDLR by genetic knockout resulted in 62% reduction in* T. cruzi *infection, which suggests LDLR is essential for* T. cruzi *cell invasion process (Figure 1(c)). Furthermore, upregulation of LDLR expression was also seen in the heart of* T. cruzi *infected CD1 mice [83]. Moreover, in* Toxoplasma gondii* infection, LDLR functions to uptake LDL particles and support intracellular parasite growth [84]. It is recently demonstrated that* T. cruzi *interaction with LDL receptor leads to the increased accumulation of LDL-cholesterol in host tissue in both acute and chronic CD [85].

Alterations in the micro- and macrovascular circulations and atherosclerosis-like symptoms are commonly seen in cardiomyopathic patients [86, 87]. Bestetti et al. reported that* T. cruzi *infection in combination with a high cholesterol diet can induce early symptoms of atherosclerosis in mice [88, 89]. LDL and LDLR were implicated extensively in atherosclerosis pathology and progression. It is known that LDL particles are transported across the endothelium and become trapped in the matrix of arterial wall cells, which leads to the production of highly cytotoxic oxidized LDL and subsequently activates inflammatory pathways, such as NF*κ*B [90]. The interaction of* T. cruzi *with LDLR may increase host susceptibility to atherosclerosis and arterial pathology.

In addition to the parasite interaction with LDL and LDLR,* T. cruzi *also interacts with HDL (originally named cruzin in* T. cruzi *research [91]), the major component of the reverse cholesterol transport pathway. HDL is a complex, multistructured particle consisting of two layers of phospholipids that are held together by two molecules of apolipoprotein A-I (Apo A-I). The main function of HDL is to remove excess cholesterol from peripheral tissues and return it to the liver for storage and excretion [92]. Other functions of HDL also include inhibiting LDL oxidation, platelet aggregation and coagulations, and endothelial inflammation, as well as promoting endothelial nitric oxide production and prostacyclin bioavailability [93, 94].

Similar to LDL-*T. cruzi *interaction, HDL was shown to bind to and inhibit* T. cruzi *trypomastigotes* trans*-sialidase activity [11, 95]. Interestingly, this interaction is specific for* T. cruzi *and was not found in* Trypanosoma rangeli, *an infectious agent nonpathogenic to human hosts.* T. cruzi *and* T. rangeli *overlap geographically, share antigenic protein, and are able to infect the same triatominae vector and vertebrate hosts. HDL inhibition of* T. cruzi trans*-sialidase functions in a dose-dependent manner through a reversible noncompetitive mechanism [95]. Maximum association between HDL and* T. cruzi trans*-sialidase occurs in less than 5 min and lasts more than 120 min [11]. More importantly, HDL inhibition of* T. cruzi trans*-sialidase enhances parasite infection* in vitro* [10]. Recently, Weizong et al. have discovered similar interaction between Apo A-I and Dengue virus. The research group showed that Apo A-I is associated with the virus particles and preincubation of dengue virus with HDL enhances viral infection through a scavenger receptor-BI- (SR-BI-) mediated mechanism [96]. These findings may also provide a possible mechanism for the enhancement of* T. cruzi *infection by HDL (Figure 1(e)). Furthermore, our research has shown that, during the intracellular amastigote stage of infection, groups infected in the presence of HDL had lower number of intracellular parasites than groups without HDL (Q. Miao & M. Ndao, personal communication). It is possible that HDL inhibition of* T. cruzi trans*-sialidase led to the decreased rate of trypomastigotes escaping from the parasitophorous vacuole and delaying the process of trypomastigote transformation [97].

In the* T. cruzi *epimastigote form, HDL may also be endocytosed and function as nutritional supply [10]. HDL endocytosis was first observed in* Trypanosoma brucei brucei *(*T. b. brucei*).* T. brucei* (African trypanosome) is closely related to* T. cruzi *(American trypanosome) in evolutionary lineage and shares a high level of biological resemblance. In the interaction of HDL with* T. brucei*, HDL is named trypanolytic factor (TLF), because endocytosis of certain HDL subspecies, which contain haptoglobin-related protein (Hpr, TLF-1 [98]) and apolipoprotein L-I (Apo L-I, TLF-2 [99]), causes lysis of* T. b. brucei *and protects mammalian hosts from infection [100]. However,* T. cruzi *has developed resistance to TLFs. The exact mechanism of this resistance is currently unknown.

The interaction between HDL and* T. cruzi *was recently reinforced by the discovery that the major structural component of HDL, apolipoprotein A-I (Apo A-I, full-length 28.1 kDa), is truncated into fragments (24.7, 13.6, 10.3, and 9.3 kDa) in sera of* T. cruzi *infected patients [101]. Apo A-I (243 amino acids) accounts for ~75% of HDL protein content [102]. Both the N- and the C-termini of Apo A-I are involved in lipid binding functions [103–105]. The central domain of the Apo A-I protein is involved in the activation of lecithin-cholesterol acyltransferase (LCAT), which is responsible for the esterification and storage of cholesterol within HDL particles [106]. Minor changes in the Apo A-I amino acid sequence or structure could seriously affect HDL function [107]. Therefore, Apo A-I truncation seen in* T. cruzi *infection may contribute to the dysregulation of host lipid metabolism. The effect of this dysregulation needs to be further investigated. However, the unique truncation pattern seen in these patients has high discriminatory power between infected and uninfected patients and can be used as* T. cruzi *diagnostic biomarkers [101, 108, 109].

Our research has revealed that the series of Apo A-I truncations was facilitated by the major cysteine protease of* T. cruzi*, cruzipain [56], which is also known as GP 57/51 or cruzain. This protease which belongs to the mammalian papain superfamily is known to cleave immunoglobulin class G proteins [110, 111]. Cruzipain has an essential function in the invasion and survival processes of* T. cruzi *and is expressed in all developmental stages of the parasite life cycle [110]. At each stage, cruzipain is differentially located within the parasite to carry out stage specific functions [112, 113]. In the* T. cruzi *trypomastigote form, cruzipain is located on the parasite surface, flagellar pocket, and lysosome-like structure [114, 115].

**Figure 2:**
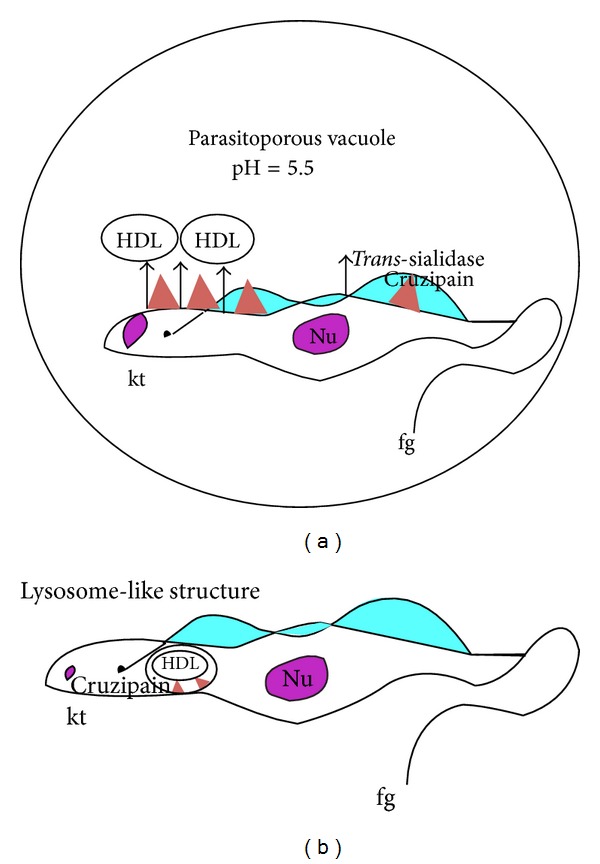
Possible mechanisms of* T. cruzi *cruzipain cleaving Apo A-I in HDL.* T. cruzi *cruzipain is expressed in the parasitic surface as well as in the lysosomal-like structure/reservosome. Both cruzipain fractions are required to produce the full Apo A-I truncation profile seen in* T. cruzi *infected human patients. This implies that Apo A-I within the HDL complex may be (a) truncated during the infection process on the parasitic surface and also (b) endocytosed by* T. cruzi* and processed in the reservosome for possible lipid utilization.

It was also shown that cruzipain was only able to cleave Apo A-I at an acidic pH, which suggests that the cleavage may take place within acidic environments. Furthermore, cruzipain from parasite surface (Figure 2(a)) and cruzipain within the lysosome-like structure (Figure 2(b)) are both required in order to produce the truncation pattern [56]. It is interesting to note that the localization of cruzipain highly resembles that of* trans*-sialidase. Therefore, it is possible that HDL is both endocytosed by trypomastigotes and bound to the surface of the parasite via* trans*-sialidase. During the infection process, the parasite bound HDL is cleaved by cruzipain in the acidic parasitophorous vacuole.

With the emerging evidence, it is becoming obvious that* T. cruzi *exploits the complex cholesterol transport system via a variety of molecules such as LDL, LDL-R, and HDL. The results of these interactions seem to all lead to the establishment of* T. cruzi *infection and Chagas disease chronicity. The impact of these relationships on host lipid metabolism is yet to be investigated.

## 5. Conclusion

Host lipid metabolism is a intricate system involving a wide range of factors. It interacts with other energy metabolic systems as well as the immune system. The role of host lipid metabolism in response to infectious agents is drawing increasing attention. This review may aid in deeper understanding of* T. cruzi *interacting with host lipid metabolism with a more systematic approach, as well as the role of lipids in* T. cruzi *pathogenesis. We have clearly illustrated that* T. cruzi *interacts with several specific factors in host lipid metabolism. Further research in these interactions and the role of lipids in* T. cruzi *pathogenesis will be highly useful in the future.
